# Gut microbiome predictors of *Escherichia coli* sequence type 131 colonization and loss

**DOI:** 10.1016/j.ebiom.2023.104909

**Published:** 2023-12-13

**Authors:** Daniel E. Park, Maliha Aziz, Benjamin J. Koch, Kelsey Roach, Connie Clabots, James R. Johnson, Lance B. Price, Cindy M. Liu

**Affiliations:** a Department of Environmental and Occupational Health, Milken Institute School of Public Health, George Washington University, Washington, DC, 20052, USA; b Center for Ecosystem Science and Society, Northern Arizona University, P.O. Box 5620, Flagstaff, AZ, 86011, USA; c Department of Biological Sciences, Northern Arizona University, 617 S Beaver St., Flagstaff, AZ, 86011, USA; d Minneapolis Veterans Affairs Health Care System, 1 Veterans Dr, Minneapolis, MN, 55417, USA; e Department of Medicine, University of Minnesota, 401 East River Parkway, VCRC 1st, UK Floor, Suite 131, Minneapolis, MN, 55455, USA

**Keywords:** Gut microbiome, *Escherichia coli*, ST131, *H*30R, *Alistipes*, *Collinsella*

## Abstract

**Background:**

*Escherichia coli* sequence type 131 (ST131), specifically its fluoroquinolone-resistant *H*30R clade (ST131-*H*30R), is a global multidrug-resistant pathogen. The gut microbiome's role in ST131-*H*30R intestinal carriage is undefined.

**Methods:**

Veterans and their household members underwent longitudinal fecal swab surveillance for ST131 in 2014–2018. The fecal microbiome was characterized by 16S rRNA qPCR and sequencing. We evaluated associations between ST131-*H*30R carriage and gut microbiome at baseline by random forest models to identify the most informative gut bacterial phyla and genera attributes for ST131 and ST131-*H*30R carriage status. Next, we assessed longitudinal associations between fecal microbiome and ST131-*H*30R carriage using a mixed-effects logistic regression with longitudinal measures.

**Findings:**

Of the 519 participants, 78 were carriers of ST131, among whom 49 had ST131-*H*30R. At the baseline timepoint, *H*30R-positive participants had higher proportional abundances of Actinobacteria phylum (mean: 4.9% vs. 3.1%) than ST131-negative participants. *H*30R-positive participants also had higher abundances of *Collinsella* (mean: 2.3% vs. 1.1%) and lower abundances of *Alistipes* (mean: 2.1% vs. 2.6%) than ST131-negative participants. In the longitudinal analysis, *Collinsella* abundance correlated positively with ST131-*H*30R carriage status and negatively with the loss of ST131-*H*30R. Conversely, *Alistipes* corresponded with the loss and persistent absence of ST131-*H*30R even in the presence of a household exposure.

**Interpretation:**

Abundances of specific fecal bacteria correlated with ST131-*H*30R carriage, persistence, and loss, suggesting their potential as targets for microbiome-based strategies to reduce carriage of ST131-*H*30R, a significant risk factor for invasive infections.

**Funding:**

This work was supported in part by 10.13039/100000060National Institute of Allergy and Infectious Diseases of the National Institutes of Health under award numbers R21AI117654 and UM1AI104681 and the 10.13039/100006379Office of Research and Development, 10.13039/100000738Department of Veterans Affairs. The content is solely the responsibility of the authors and does not necessarily represent the official views of the National Institutes of Health or the Department of Veterans Affairs.

**Research in context**

**Evidence before this study**

*Escherichia coli* sequence type 131 and its fluoroquinolone-resistant *H*30R subclone lineage (ST131-*H*30R) is a leading cause of antimicrobial resistant *E. coli* infections globally. ST131-*H*30R is commonly carried in the gut as an opportunistic pathogen before subsequent infection of the colonized host. Gut microbiome determinants of ST131-*H*30R carriage are unclear and have not been evaluated in longitudinal studies.

**Added value of this study**

Using a large cross-sectional study, we used random forest models to identify bacteria associated with ST131-*H*30R carriage. Participants with ST131-*H*30R exhibited higher proportional abundance of Actinobacteria and *Collinsella*. Conversely, *Alistipes* was more common in participants without ST131-*H*30R carriage. These gut microbiome patterns were confirmed in an extensive longitudinal study of individuals and their household members, where *Collinsella* was present more frequently among persistent ST131-*H*30R carriers, while *Alistipes* increased in conjunction with loss of ST131-*H*30R carriage.

**Implications of all the available evidence**

Our data indicated that multiple gut bacteria corresponded with *E. coli* ST131-*H*30R gut carriage, persistence, and loss. *Collinsella* has been previously linked with gut inflammation and permeability, whereas *Alistipes* has been linked with anti-inflammatory effects and has been reported as inversely correlated with *Escherichia* carriage. This study supports these findings using longitudinal analyses, and extends them specifically to ST131-*H*30R, showing higher *Collinsella* abundance alongside persistent ST131-*H*30R carriage, and a significant increase in *Alistipes* abundance in conjunction with persistent loss of ST131-*H*30R. These gut microbiome taxa (*Alistipes*, *Collinsella*), or the pathways they represent, may provide targets for therapeutic interventions through which to reduce or prevent ST131-*H*30R carriage, thereby decreasing transmission and infections from this critical multidrug-resistant opportunistic pathogen.

## Introduction

*Escherichia coli (E. coli)* sequence type 131 (ST131), especially its recently emerged *H*30R subclone and the two main clonal subsets within *H*30R—*H*30R1 and *H*30Rx—cause a substantial portion of antimicrobial-resistant infections in the U.S. and worldwide.^1^ Both *H*30R1 and *H*30Rx are characterized by fluoroquinolone resistance. Additionally, many *H*30R strains—especially within *H*30Rx—are co-resistant to multiple other antimicrobial agents, most notably advanced beta-lactams, due to plasmidic or chromosomal extended-spectrum beta-lactamases and, increasingly, carbapenemases.

Gut colonization by ST131-*H*30R significantly increases the risk for subsequent infection.^2^ Individuals can become colonized by antibiotic-resistant *E. coli* via horizontal transmission, such as through exposures from international travel,^3^ within households,^4^ or in the healthcare setting.^5^ A key feature of ST131-*H*30R—including the *H*30R1 and *H*30Rx subsets—that underlies its pandemic emergence is its ability to stably colonize the human gastrointestinal tract,^2^^,^^6^ which is mediated, at least in part through bacterial factors such as FimH,^7^^,^^8^ fluoroquinolone resistance,^9^ and accessory traits such as iron uptake systems and protectins.^6^ However, not all individuals exposed to known carriers of ST131-*H*30R become colonized.^10^^,^^11^

Studies of probiotics have found that certain gut microbiome features are associated with failed probiotic engraftment, suggesting that microbiome composition can determine whether colonization occurs after exposure to a new bacterium.^12^ This potential microbiome-mediated resistance to gut bacterial colonization may extend to opportunistic pathogens such as ST131-*H*30R, which if true could lead to probiotic solutions to protect against ST131-*H*30R colonization. Previous studies, including a longitudinal study of 27 Dutch nursing home residents,^13^ have examined the association of gut microbiome composition and host colonization by fluoroquinolone-resistant *E. coli*^14^ or multidrug-resistant organisms.^15^ However, the influence of gut microbiome on gut colonization by the major pandemic subclone ST131-*H*30R is unknown.

Our main study objective was to determine how the gut microbiome correlates with carriage, persistence, and loss of ST131-*H*30R. We identified and followed the members of households within which at least one individual carried fluoroquinolone-resistant *E. coli* and/or ST131. Using the combination of a cross-sectional study of 519 participants and a nested longitudinal household study of 141 participants, we determined how gut microbiome composition correlates with the temporal dynamics of ST131 carriage.

## Methods

### Ethics and sample collection

Fecal swabs from consenting subjects were collected according to a protocol approved by the University of Minnesota Institutional Review Board (1412M58401), Minneapolis VA Health Care System IRB (4490-A), and George Washington University IRB (121508). As described elsewhere,^6^^,^^16^ study subjects included military veterans under care at the Minneapolis Veterans Affairs Medical Center (MVAMC) and their household members. Veterans were recruited prospectively (2014 through 2018) by sending invitations for study participation to all newly discharged MVAMC inpatients and randomly selected outpatients. Veterans who agreed to participate were encouraged to refer all available adult and child household members and pets. Swabs were mailed to the research laboratory at room temperature in commercial transport medium, along with basic demographic information. Other clinical data were not obtained.

To assess persistent ST131 carriage, subjects whose initial fecal swab yielded fluoroquinolone-resistant *E. coli* and/or ST131 (as detected using the below-described methods) were offered serial fecal sampling, along with their household members. Serial sampling was done monthly for six months, then every three months, until the first of the following occurred: the end of the study period, the subject/household declined further follow-up, or on two consecutive sampling occasions no household member yielded the household's initial strain of interest.

### Culture methods

In the research laboratory, fecal swabs provided by study participants were screened for fluoroquinolone-resistant *E. coli* by selective culture (using Tergitol-7 agar plates with and without ciprofloxacin 4 μg/mL), for overnight incubation at 37 °C. Individual colonies of presumptive *E. coli*—i.e., lactose-positive colonies with a characteristic *E. coli* morphology—were assessed for species identity using indole and citrate phenotype (indole-positive, citrate-negative).

### Molecular characterization of *E. coli* isolates

We used standard protocols for detection of fluoroquinolone-resistant ST131-*H*30 isolates using highly sensitive and specific established PCR-based assays, as previously reported.^6^^,^^16^ In brief, up to 10 presumptive *E. coli* colonies per plate were screened for clonality by using random amplified polymorphic DNA (RAPD) analysis. Using duplicate boiled lysates for template DNA and relevant positive and negative controls, one representative colony per unique RAPD profile per sample was screened for ST131 by PCR and ST131 isolates were screened to identify the *H*30 and *H*30Rx subclones. Fluoroquinolone-resistant ST131-*H*30 isolates were classified as ST131-*H*30R, and *H*30R isolates that tested negative for *H*30Rx were classified as *H*30R1.^16^

### *ST131-*H*30R carriage pattern categorization*

Based on their *E. coli* strain carriage pattern, we assigned participants to one of five categories. These included: 1) sustained positive (i.e., participants with ST131-*H*30R at all time points); 2) exposed, sustained negative (i.e., participants exposed to a ST131-*H*30R positive household member but who remained ST131-*H*30R negative); 3) sustained loss (i.e., participants with at least one ST131-*H*30R isolate, followed by at least two samplings without ST131-*H*30R, and who never re-acquired ST131-*H*30R); 4) unexposed negative (i.e., participants without ST131-*H*30R and not exposed to a household member with ST131-*H*30R); and 5) other (i.e., participants not falling into the above four categories).

### Sample processing and microbiome characterization

DNA isolation and purification were performed using the collected fecal swabs. Total DNA was extracted from 200 μl of undiluted swab eluent using the MagAttract PowerMicrobiome DNA/RNA kit (Qiagen Inc., Valencia, CA, USA) and eluted into 100 μl of elution buffer and stored at −80 °C until analysis.

The prevalence and proportional abundance of specific bacterial taxa in each fecal swab were characterized by sequencing the 16S rRNA gene V3–V4 region as described in Fadrosh et al.^17^ using MiSeq Reagent Kit v3 (600-cycle) (Illumina Inc., San Diego, CA). A negative-extraction control (NEC) was included with each batch of extraction and sequenced in order to assess for cross-contamination. No-template controls (NTCs) and positive-template controls (PTCs) were included to assess for cross-contamination and verify PCR performance.

An in-house pipeline built with published tools was used for amplicon data processing. Briefly, primers were clipped by cutadapt v2.4^18^ and resultant sequences quality-trimmed using Trimmomatic v0.39.^19^ then processed with DADA2 v1.10^20^ to identify amplicon sequence variants (ASVs). ASVs were queried using BLAST v.2.2.30+^21^ with cut off values of 99% nucleotide identity and 100% query coverage to remove bloomed ASVs^22^ (https://github.com/knightlab-analyses/bloom-analyses/blob/master/data/newbloom.all.fna). After removing bloom ASVs, the remaining ASVs were classified at 80% bootstrap confidence level using the Naïve Bayesian Classifier (v.2.12).^23^ Additional details can be found at https://github.com/araclab/mb_analysis.

Samples with under 1000 reads were excluded from analysis. After filtering out rare taxa (any genera accounting for <0.0025% of the entire dataset), the resultant sequencing data were used to calculate the prevalence and the proportional abundance of each taxon for each sample (i.e., Number of 16S rRNA gene sequences assigned to a taxon divided by the total number of 16S rRNA sequences). Data from this study is available at SRA project number PRJNA838578.

### Comparative gut microbiome analysis

We first used cross-sectional data to identify gut microbiome features that are associated with ST131-*H*30R carriage, then used longitudinal data to determine how the identified gut microbiome features correlate with ST131-*H*30R carriage and loss. Samples from pets were excluded from all analyses. Primary analyses focused on comparing ST131-negative participants to *H*30R-positive participants, given the clinical ambiguity of non-*H*30R ST131 strain carriage. Supplemental analyses included comparisons across four mutually exclusive carriage subcategories (ST131-negative, non-*H*30R ST131, ST131-*H*30R1, ST131-*H*30Rx).

The cross-sectional study included, for ST131 carriers, the first ST131-positive sample and, for ST131 non-carriers, the first sample overall, which by definition was ST131-negative. Alpha-diversity was measured using the Shannon diversity index. Among bacterial phyla with ≥10% prevalence (i.e., proportion of subjects positive), we identified phyla associated with ST131-*H*30R carriage using analyses as described below.

We next used longitudinal data to identify gut bacterial taxa associated with ST131-*H*30R carriage. For this, we considered three sets of candidate genera. The first set comprised genera that were members of phyla identified as being associated with ST131-*H*30R carriage in the cross-sectional study and also present in at least 25% of the overall study population. The second set comprised the 10 most-informative genera, as identified by using the mean decrease in Gini values in conjunction with the largest number of inclusions in trees in random forest models. The third set comprised two genera that we regarded *a priori* as being of interest: *Bacteroides* and *Escherichia/Shigella*. For the random forest model, we selected a breakpoint for the number of taxa to be included by identifying an elbow on a scree plot based on the Gini and margin values.

We used Chi-squared tests and Wilcoxon Rank–Sum tests to identify associations between ST131-*H*30R carriage and the prevalence and proportional abundance of phyla and genera. Correlations between ST131-*H*30R carriage and the proportional abundance of phyla and genera were evaluated among longitudinal samples using a mixed effects logistic regression model, allowing for repeated measures by individual. Statistical analyses were conducted in SAS, version 9.4, and R, version 3.3.1.

### Role of the funding source

The funders had no role in study design, data collection, analysis, or interpretation, or any aspect pertinent to the study.

## Results

### Participant characteristics and ST131 carriage

Of the 519 participants, 62.8% were veterans, 31.6% were a veteran's spouse, and 5.6% were a child or non-spouse adult in the household (Table 1). The initial fecal screening by conventional culture and PCR identified 78 (15.0%) participants as carriers of ST131 and/or fluoroquinolone-resistant *E. coli.* All members of households with an ST131 *E. coli* carrier were invited to undergo longitudinal surveillance; an average of 1.6 participants were enrolled per household. In total, we followed longitudinally 141 participants, including 44 of 49 *H*30R-positive participants (mean, 8.5 samples each [SD = 3.8]) and 73 of 441 ST131-negative participants (mean, 5.5 samples each [SD = 2.3]).

**Table 1:** Demographic and other characteristics of participants.

Baseline characteristics	No. (column %)			
	Non-ST131 n = 441	Any ST131 n = 78	ST131	
			Non-*H*30R n = 29	*H*30R n = 49
**Participant**				
Veteran (patient)	279 (63.3%)	47 (60.3%)	18 (62.1%)	29 (59.2%)
Child	17 (3.9%)	2 (2.6%)	1 (3.5%)	1 (2.0%)
Spouse	136 (30.8%)	28 (35.9%)	10 (34.5%)	18 (36.7%)
Other Adult	9 (2.0%)	1 (1.3%)	0 (−)	1 (2.0%)
**Patient type**				
Inpatient	186 (42.2%)	25 (32.1%)	14 (48.3%)	12 (24.5%)
Outpatient	255 (57.8%)	53 (68.0%)	15 (51.7%)	37 (75.5%)
**Longitudinal visits**				
≥2 longitudinal visits	84 (19.0%)	57 (73.1%)	22 (75.9%)	36 (73.5%)

### Prevalence and dynamics of intestinal *E. coli* and ST131

According to genus-level sequencing data, at baseline 463 (89.2%) of the 519 study participants carried *Escherichia*. The proportional abundance of *Escherichia* at baseline ranged from 0% to 92.1% (mean 18.8%, SD 19.1) among the 519 participants.

**Fig. 1:**
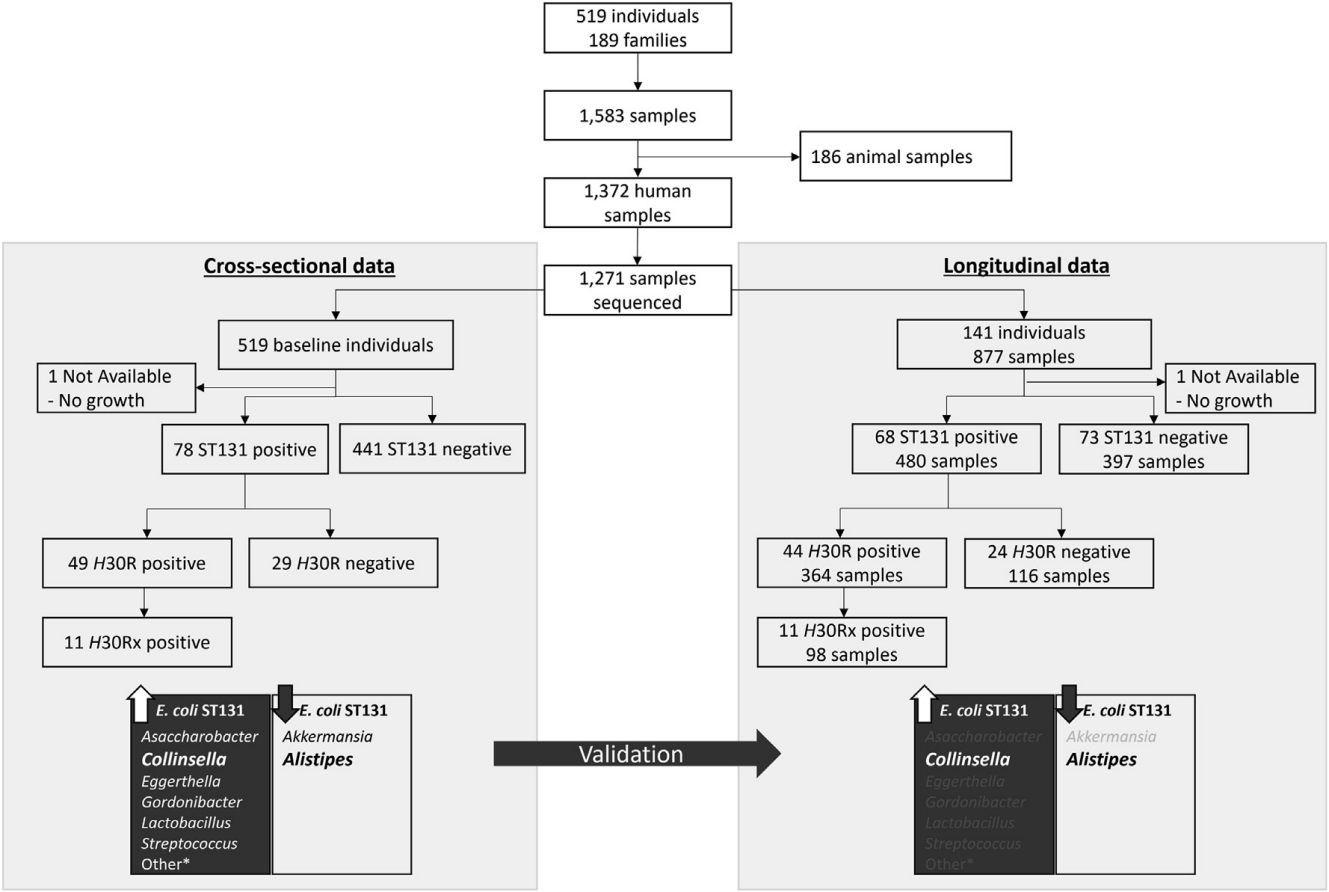
**Participant flow for inclusion into cross-sectional and longitudinal analyses, for the ST131, *H*30, and *H*30R categories**. Flowchart for the cross-sectional and longitudinal data, including characterization of the number of families, individuals, samples, and molecular characterization of *E. coli* isolates. High-level associations between taxa and *E. coli* ST131 carriage are provided at the bottom, with cross-sectional association findings being validated in a subset of individuals with available longitudinal data.

According to PCR testing, 78 (15.0%) of the 519 participants had ST131 in one or more samples, including 49 (9.2%) with *H*30R, among which 38 (7.1%) were *H*30R1 and 11 (2.1%) were *H*30Rx (Fig. 1). Of the 141 participants with longitudinal data, 25 (18%) were persistently *H*30R-positive (i.e., sustained positives), 11 (8%) were persistently *H*30R-negative despite household exposure (i.e., exposed, sustained negatives), 14 (10%) were *H*30R-positive, then persistently *H*30R-negative (i.e., sustained loss), 62 (44%) were *H*30R-negative in the absence of household exposure (unexposed negatives), and 27 (19%) exhibited other patterns.

### Cross-sectional analysis for gut microbiome characteristics associated with ST131-*H*30R carriage

To identify gut bacterial taxa associated with carriage of ST131-*H*30R, we first performed a cross-sectional analysis involving all 519 participants, focusing initially on phyla, then genera. Alpha diversity was similar across all ST131 sub-categories (non-*H*30R, *H*30R, and *H*30Rx; p = 0.95), but was significantly lower when comparing ST131-negative individuals compared with ST131-positive individuals (p = 0.012, Supplemental Figure S4).

At the phylum level, ST131-*H*30R carriage was associated with both a higher proportional abundance of Actinobacteria and, conversely, a lower proportional abundance of Verrucomicrobia. Specifically, *H*30R-positive participants had a significantly higher proportional abundance of Actinobacteria (mean 4.9%, SD 4.1), as compared to ST131-negative participants (mean 3.1%, SD 4.6) (p = 0.002) (Table 2). Additionally, *H*30Rx-positive participants had the highest abundance of Actinobacteria (mean 6.7%, SD 4.0), whereas participants with no detectable *Escherichia* had the lowest abundance (mean 1.3%, SD 1.6) (Supplemental Table S1, Supplemental Figure S1). By contrast, *H*30R-positive participants had a significantly lower proportional abundance of Verrucomicrobia (mean 0.3%, SD 1.0) than did ST131-negative participants (mean 1.3%, SD 3.4) (p = 0.04).

**Table 2:** Prevalence and mean proportional abundance of genera in cross-sectional analysis.

Phylum or *genus*	Random forest^a^	Prevalence^b^, column percent				p value		Proportional abundance^c^, mean (SD)				p value	
		A. non-ST131	B. any ST131	C. ST131, non-*H*30R	D. ST131, *H*30R	A vs B	A v D	A. non-ST131	B. any ST131	C. ST131, non-*H*30R	D. ST131, *H*30R	A vs B	A v D
		N = 441	N = 78	N = 29	N = 49			N = 441	N = 78	N = 29	N = 49		
**Actinobacteria**		94	96	100	94	0.51	0.90	3.1 (4.6)	4.7 (3.9)	4.3 (3.6)	4.9 (4.1)	**<0.001**	**0.001**
*Asaccharobacter*^d^	X	25	46	48	45	**<0.001**	**0.004**	0.1 (0.1)	0.1 (0.2)	0.1 (0.2)	0.1 (0.2)	**<0.001**	**0.008**
*Bifidobacterium*		62	71	72	69	0.15	0.30	0.9 (2.4)	0.8 (1.2)	0.8 (1.2)	0.7 (1.3)	0.32	0.78
*Collinsella*	X	67	82	90	78	**0.007**	0.12	1.1 (2.5)	2.1 (2.5)	1.9 (2.3)	2.3 (2.7)	**<0.001**	**0.001**
*Eggerthella*	X	47	62	72	55	**0.02**	0.31	0.2 (0.4)	0.6 (1.1)	0.7 (1.0)	0.6 (1.2)	**0.001**	0.18
*Gardnerella*	X	7	26	38	18	**<0.001**	**0.006**	0.0 (0.3)	0.2 (1.0)	0.1 (0.3)	0.3 (1.3)	**<0.001**	**0.004**
*Gordonibacter*		32	54	55	53	**<0.001**	**0.003**	0.3 (1.0)	0.5 (1.2)	0.5 (1.2)	0.5 (1.2)	**<0.001**	**0.01**
**Bacteroidetes**		100	100	100	100	1.00	1.00	30.5 (15.7)	27.6 (13.0)	24.5 (13.0)	29.4 (12.8)	0.20	0.78
*Alistipes*	X	88	82	90	78	0.15	**0.04**	2.6 (3.8)	1.8 (2.9)	1.2 (1.4)	2.1 (3.5)	**0.01**	0.08
*Bacteroides*		100	100	100	100	0.55	0.64	23.9 (14.7)	22.2 (11.8)	20.4 (11.3)	23.3 (12.1)	0.52	0.78
**Firmicutes**		100	100	100	100	0.55	0.64	39.7 (18.3)	40.9 (14.3)	39.2 (16.5)	41.9 (12.9)	0.35	0.22
*Enterococcus*	X	43	53	55	51	0.10	0.26	0.8 (3.1)	2.8 (8.0)	4.5 (10.5)	1.9 (6.0)	**0.03**	0.17
*Lactobacillus*	X	33	65	62	67	**<0.001**	**<0.001**	0.3 (1.2)	1.3 (3.3)	1.0 (3.0)	1.5 (3.5)	**<0.001**	**<0.001**
*Streptococcus*	X	68	88	97	84	**<0.001**	**0.02**	1.2 (3.9)	2.0 (4.5)	1.5 (2.0)	2.3 (5.4)	**<0.001**	**0.001**
*Weissella*	X	2	9	14	6	**0.002**	0.11	0.0 (0.3)	0.0 (0.2)	0.0 (0.1)	0.0 (0.2)	**0.003**	0.10
**Fusobacteria**		13	14	14	14	0.78	0.79	0.2 (1.2)	0.1 (0.8)	0.1 (0.2)	0.2 (1.0)	0.87	0.85
**Lentisphaerae**		10	5	0	8	0.16	0.65	0.0 (0.1)	0.0 (0.0)	–	0.0 (0.0)	0.14	0.61
**Proteobacteria**		98	100	100	100	0.20	0.31	24.8 (20.4)	25.5 (17.5)	31.3 (18.6)	22.0 (16.0)	0.35	0.74
*Escherichia*		88	99	100	98	**0.003**	**0.03**	18.7 (19.6)	19.5 (16.0)	22.8 (16.8)	17.6 (15.4)	0.07	0.37
*Oxalobacter*^d^	X	4	12	3	16	**0.004**	**<0.001**	0.0 (0.0)	0.0 (0.0)	0.0 (0.0)	0.0 (0.0)	**0.004**	**<0.001**
**Synergistetes**		13	13	0	20	0.98	0.15	0.1 (0.3)	0.4 (3.0)	–	0.6 (3.7)	0.95	0.16
**Verrucomicrobia**		52	38	34	41	**0.048**	0.22	1.3 (3.4)	0.3 (0.9)	0.2 (0.8)	0.3 (1.0)	**0.003**	**0.04**
*Akkermansia*		52	38	34	41	**0.03**	0.14	1.3 (3.4)	0.3 (0.9)	0.2 (0.8)	0.3 (1.0)	**0.005**	**0.02**

Bold denotes *p* < 0.05.

a X indicates the taxon was selected as one of the top-10 most informative genera for predicting ST131 carriage using the random forest model.

b Percent of participants in a group with a given taxon.

c Mean proportional contribution of each taxon to each participant's gut microbiome (proportional abundance for a given sample was calculated as the number of reads of the given taxon divided by the total number of reads in that sample).

d A genetic near neighbor of the taxon.

By contrast with Actinobacteria and Verrucomicrobia, the phyla Bacteroidetes, Firmicutes, and Proteobacteria occurred in nearly all participants, and did not differ significantly in prevalence or abundance by ST131 status or ST131 subclone carriage (not shown). Firmicutes was the most proportionally abundant of these phyla (mean 39.9%, SD 17.8), followed by Bacteroidetes (mean 30.1%, SD 15.4) and Proteobacteria (mean 24.9%, SD 20.0).

### Cross-sectional analysis for genera and species associated with ST131-*H*30R carriage

At the genus level, although multiple genera were positively associated with *H*30R carriage, only *Alistipes* was negatively associated with *H*30R carriage. Specifically, of the genera examined, *Gardnerella, Gordonibacter, Lactobacillus, Streptococcus*, and a phylogenetic near neighbor of *Asacharobacter* all exhibited a higher prevalence and greater proportional abundances in association with carriage of ST131 and *H*30R, and *Collinsella*, *Eggerthella,* and *Weissella* exhibited similar association with carriage of ST131 (but not *H*30R).

By contrast, *Alistipes* and *Akkermansia* were negatively associated with *H*30R carriage. Specifically, as compared with *H*30R-positive participants, ST131-negative participants had a higher prevalence or proportional abundance of *Alistipes* (p = 0.04 and p = 0.08, respectively), and a higher proportional abundance of *Akkermansia* (p = 0.02), although a similar prevalence of *Akkermansia* (p = 0.14). Species-level associations are provided in supplemental analyses for *Alistipes*, along with the three most-prevalent genera associated with ST131 carriage (*Collinsella, Lactobacillus, Streptococcus*) (Supplemental Table S3).

### Longitudinal associations between genera and *H*30R carriage

We next performed longitudinal analyses of the gut microbiome, limited to the genus level, from 25 participants with either of two clinically relevant *H*30R colonization patterns: exposed, sustained negative (participants with a *H*30R-positive household member who remained *H*30R-negative for the duration of the study) and sustained loss (*H*30R-positive individuals who became *H*30R-negative during the study). On average, participants contributed 7.6 visits.

**Fig. 2:**
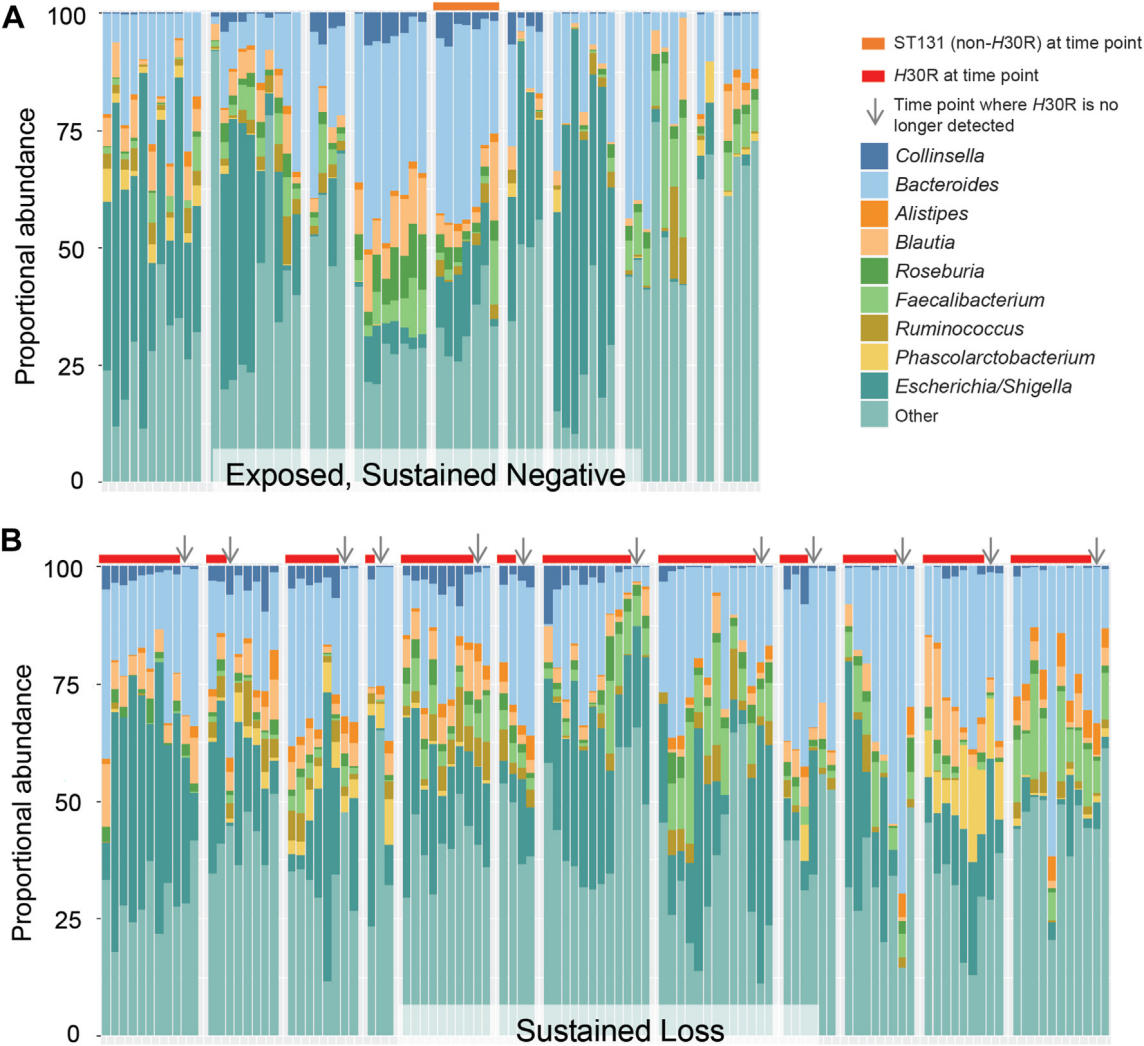
**Gut microbiota genera proportional abundance among exposed, sustained *H*30-negative (A) and sustained *H*30R loss participants (B)**. Each group of adjoined columns represents a sequential (from left to right) time series of samples from an individual participant. Columns represent the proportional abundance of genera at each time point. Colored horizontal bars above the columns represent ST131 (non-*H*30R, orange) and ST131-*H*30R (red) status over time for each participant. Arrows represent the time point where *H*30R is no longer detected for participants with sustained loss, which is generally accompanied by loss of *Collinsella*.

Findings from the cross-sectional analysis were corroborated in the longitudinal analysis by using a mixed-effects logistic regression model that controlled for intra-individual variation over time and included all genera of interest from Table 2. *Alistipes* was the only genus significantly associated with absence of *H*30R (p = 0.005), whereas *Collinsella* was the only genus associated with presence of *H*30R (p = 0.035) (Supplemental Table S2). No other taxa displayed significant associations with *H*30R carriage. Among participants with sustained loss of *H*30R, *Collinsella* decreased in median proportional abundance with loss of *H*30R, from 1.5% to 0.5% (Fig. 2). Conversely, median proportional abundance of *Alistipes* increased from 0.8% to 2.0% after loss of *H*30R. *Collinsella* and *Alistipes* median proportional abundance were both low (0.2% and 0.4%, respectively) for participants who were exposed to a *H*30R-positive family member but remained ST131-negative.

## Discussion

In this study of veterans and their household members, we found that specific phyla and genera in the gut microbiome were consistently associated with ST131 and *H*30R carriage. In cross-sectional comparisons, the phylum Actinobacteria and certain common genera, including *Lactobacillus, Streptococcus,* and *Collinsella* (a member of Actinobacteria), were associated with presence of ST131 and *H*30R. In longitudinal analyses, the genus *Collinsella* again was associated with *H*30R-positivity, whereas the genus *Alistipes* was associated with absence and loss of ST131 and *H*30R. These findings identify potential opportunities for influencing gut colonization by ST131 and its *H*30R subclone.

The *H*30R subclone of *E. coli*, which has emerged as an important multidrug-resistant pathogen, causes a significant portion of antimicrobial-resistant infections in the U.S. and worldwide.^1^^,^^24^ Although *H*30R is known to be an excellent gut colonizer,^6^ the gut microbiome features associated with acquisition, persistence, and loss of *H*30R carriage are poorly understood. To our knowledge, this study provides the first description of the bacterial phyla and genera associated with *H*30R gastrointestinal carriage. If the observed associations represent a causal effect of specific microbiota on *H*30R colonization, interventions that shift those taxa could have important public health benefits.

*Collinsella* is a member of the phylum Actinobacteria which was associated with presence of ST131 and *H*30R in both the cross-sectional and longitudinal analyses (specifically, *C. aerofaciens*; Supplemental Table S3). *Collinsella* may be an important health-relevant constituent of the gut microbiome.^25^ Presence of *Collinsella* has been associated with diverse diseases, including irritable bowel syndrome, gestational diabetes mellitus, and atherosclerosis,^26^, ^27^, ^28^ whereas increased abundance of *Collinsella aerofaciens* has been associated with rheumatoid arthritis.^29^ Low dietary fiber intake by overweight pregnant women may allow overgrowth of *Collinsella*, have pro-inflammatory effects, and confer increased susceptibility to impaired glucose tolerance.^30^ *Collinsella aerofaciens* has also been linked with increased gut permeability and inflammation, specifically with production of IL-17A, along with high levels of alpha-aminoadipic acid and asparagine.^29^ Conceivably, the associations we observed of *Collinsella* with ST131 and *H*30R may be due to *Collinsella*-mediated modulation of the host inflammatory and immune systems in an ST131-favoring manner. If so, this would make *Collinsella* a potential intervention target. Alternatively, these associations may reflect nutritional or other exposures that jointly favor *Collinsella,* ST131, and *H*30R. Identification of such causal factors could have even broader health benefits. Evaluating causal mechanisms may require co-culture models, metagenomic, or meta-transcriptomic approaches.

By contrast with *Collinsella*, the genus *Alistipes* was negatively associated with *H*30R prevalence and ST131 proportional abundance. It has contrastingly been implicated as a risk factor for certain conditions, such as hypertension,^31^ but protective against others, including atrial fibrillation, cardiovascular disease,^32^, ^33^, ^34^ ulcerative colitis,^35^ and liver fibrosis.^36^ Studies have linked *Alistipes* with anti-inflammatory effects in the gut.^37^^,^^38^ These anti-inflammatory effects may be mediated by production of the short-chain fatty acids propionate and acetate,^39^^,^^40^ or through^37^^,^^38^ other immuno-modulatory mechanisms, leading to reduced inflammation in the gut and other organ systems.^41^^,^^42^ As with *Collinsella*, hypothesis testing regarding possible inflammation-mediated mechanisms for the observed relationship between *Alistipes* and *H*30R may be worthy of future consideration given the tremendous public health importance of *H*30R colonization.

Interestingly, multiple gut microbiome studies have observed an inverse correlation between *Alistipes* and both *Escherichia* and *Streptococcus*,^32^^,^^34^^,^^42^ Conceivably, reductions in *Alistipes* may contribute independently to both disease pathogenesis and ST131 and *H*30R carriage. Further evaluations of interactions between these key taxa may have implications for inflammation and disease and may elucidate potential targets for reductions in ST131-*H*30R colonization such as targeted probiotics and diet modifications.

The study had limitations. First, the limited demographic and clinical data precluded adjustment for potentially important exposures such as antibiotic use, diet, recent travel, and probiotic use; absence of these important factors in the available data limit our ability to infer causal associations. Second, fecal swabs underwent extended room temperature exposure between collection and processing. To address this, we used bioinformatics methods to reduce distortions from potential in vitro blooms and limited our analyses to prevalence and proportional abundance, which are less affected by overgrowth of specific taxa. The consistency of findings between prevalence and proportional abundance measures, and also between cross-sectional and longitudinal analyses, support validity of the findings with regards to both sensitivity of detecting ST131-*H*30R isolates and also conclusions regarding *Alistipes*, *Collinsella*, and other taxa of interest. Third, the study population was restricted temporally, geographically, and socio-culturally; other populations might yield different results. Fourth, fecal swabs may not accurately reflect the total gut microbiota. Fifth, while species-level data for the most prevalent genera associated with ST131-*H*30R were evaluated in supplemental cross-sectional analyses (Supplemental Table S3), we did not apply the longitudinal model due to concerns of sample size and overfitting with the large number of species. Sixth, the resolution of 16S rRNA gene amplicon sequencing may be limited to the genus level due to high similarity in the 16S rRNA gene for some closely related species. Lastly, due to sample size limitations, our primary analyses focused on comparing ST131 to no ST131 carriage, rather than across all possible subclone categories. Different *E. coli* subclones may exhibit differing niche adaptive abilities,^43^ setting the stage for niche-specific competition and behavior.^44^

In summary, gastrointestinal carriage of ST131, and specifically *H*30R, a leading antimicrobial-resistant pathogen, is associated with presence and abundance of *Collinsella* and certain other genera, whereas absence and loss of *H*30R are associated with *Alistipes*. If these associations involving the gut microbiota represent causal pathways that reduce ST131 carriage, bacteria negatively associated with ST131 carriage may be useful directly as a probiotic species, or indirectly via a prebiotic or other dietary manipulations that may promote their growth and thereby protect against ST131 colonization. Conversely, bacteria positively associated with ST131 carriage may be potential targets for vaccines or narrow-spectrum therapeutics such as bacteriophages that aim to mitigate an important public health threat.

## Contributors

JRJ, LBP, CML conceived and initiated the study. JRJ, BJK, CC curated the data. BJK, KR, DEP, MA, CML conducted the statistical analysis. JRJ, CC supervised the study. JRJ, LBP, CML, DEP, MA drafted and revised the manuscript. All authors read and approved the final manuscript.

## Data sharing statement

The dataset supporting the conclusions of this article is available at SRA project number PRJNA838578 Any additional relevant data are available upon request.

## Declaration of interests

J. R. J. has received grants or consultancies from Achaogen/Cipla, Allergan, Janssen/Crucell, Melinta, Merck, Shionogi, Syntiron, and Tetraphase, and has patent application for tests to detect specific *E. coli* strains. All other authors report no actual or potential conflicts.

## References

[1] Johnson J.R., Porter S., Thuras P., Castanheira M. (2017). The pandemic H30 subclone of sequence type 131 (ST131) as the leading cause of multidrug-resistant Escherichia coli infections in the United States (2011-2012). Open Forum Infect Dis.

[2] Tchesnokova V.L., Rechkina E., Chan D. (2020). Pandemic uropathogenic fluoroquinolone-resistant Escherichia coli have enhanced ability to persist in the gut and cause bacteriuria in healthy women. Clin Infect Dis.

[3] Langelier C., Graves M., Kalantar K. (2019). Microbiome and antimicrobial resistance gene dynamics in international travelers. Emerg Infect Dis.

[4] Valverde A., Grill F., Coque T.M. (2008). High rate of intestinal colonization with extended-spectrum-β-lactamase-producing organisms in household contacts of infected community patients. J Clin Microbiol.

[5] Hilty M., Betsch B.Y., Bögli-Stuber K. (2012). Transmission dynamics of extended-spectrum β-lactamase–producing enterobacteriaceae in the tertiary care hospital and the household setting. Clin Infect Dis.

[6] Johnson J.R., Clabots C., Porter S.B., Bender T., Johnston B.D., Thuras P. (2022). Intestinal persistence of colonizing Escherichia coli strains, especially ST131- H 30, in relation to bacterial and host factors. J Infect Dis.

[7] Sokurenko E.V., Chesnokova V., Dykhuizen D.E. (1998). Pathogenic adaptation of Escherichia coli by natural variation of the FimH adhesin. Proc Natl Acad Sci USA.

[8] Connell I., Agace W., Klemm P., Schembri M., Mărild S., Svanborg C. (1996). Type 1 fimbrial expression enhances Escherichia coli virulence for the urinary tract. Proc Natl Acad Sci USA.

[9] Johnson J.R., Urban C., Weissman S.J. (2012). Molecular epidemiological analysis of Escherichia coli sequence type ST131 (O25:H4) and bla CTX-M-15 among extended-spectrum-β-lactamase-producing E. coli from the United States, 2000 to 2009. Antimicrob Agents Chemother.

[10] Johnson J.R., Davis G., Clabots C. (2016). Household clustering of Escherichia coli sequence type 131 clinical and fecal isolates according to whole genome sequence analysis. Open Forum Infect Dis.

[11] Torres E., López-Cerero L., Morales I., Navarro M.D., Rodríguez-Baño J., Pascual A. (2018). Prevalence and transmission dynamics of Escherichia coli ST131 among contacts of infected community and hospitalized patients. Clin Microbiol Infection.

[12] Zmora N., Zilberman-Schapira G., Suez J. (2018). Personalized gut mucosal colonization resistance to empiric probiotics is associated with unique host and microbiome features. Cell.

[13] Ducarmon Q.R., Terveer E.M., Nooij S. (2021). Microbiota-associated risk factors for asymptomatic gut colonisation with multi-drug-resistant organisms in a Dutch nursing home. Genome Med.

[14] Liss M.A., Leach R.J., Rourke E. (2019). Microbiome diversity in carriers of fluoroquinolone resistant Escherichia coli. Investig Clin Urol.

[15] Araos R., Battaglia T., Ugalde J.A., Rojas-Herrera M., Blaser M.J., D'Agata E.M.C. (2019). Fecal microbiome characteristics and the resistome associated with acquisition of multidrug-resistant organisms among elderly subjects. Front Microbiol.

[16] Mohamed M., Clabots C., Porter S.B., Thuras P., Johnson J.R. (2016). Isolation and characterization of Escherichia coli sequence type 131 and other antimicrobial-resistant gram-negative Bacilli from clinical stool samples from veterans. Antimicrob Agents Chemother.

[17] Fadrosh D.W., Ma B., Gajer P. (2014). An improved dual-indexing approach for multiplexed 16S rRNA gene sequencing on the Illumina MiSeq platform. Microbiome.

[18] Martin M. (2011). Cutadapt removes adapter sequences from high-throughput sequencing reads. EMBnet J.

[19] Bolger A.M., Lohse M., Usadel B. (2014). Trimmomatic: a flexible trimmer for Illumina sequence data. Bioinformatics.

[20] Callahan B.J., McMurdie P.J., Rosen M.J., Han A.W., Johnson A.J.A., Holmes S.P. (2016). DADA2: high-resolution sample inference from Illumina amplicon data. Nat Methods.

[21] Altschul S.F., Gish W., Miller W., Myers E.W., Lipman D.J. (1990). Basic local alignment search tool. J Mol Biol.

[22] Amir A., McDonald D., Navas-Molina J.A. (2017). Correcting for microbial blooms in fecal samples during room-temperature shipping. mSystems.

[23] Wang Q., Garrity G.M., Tiedje J.M., Cole J.R. (2007). Naive Bayesian classifier for rapid assignment of rRNA sequences into the new bacterial taxonomy. Appl Environ Microbiol.

[24] Nicolas-Chanoine M.-H., Bertrand X., Madec J.-Y. (2014). Escherichia coli ST131, an intriguing clonal group. Clin Microbiol Rev.

[25] Tourlousse D.M., Sakamoto M., Miura T. (2020). Complete genome sequence of Collinsella aerofaciens JCM 10188 T. Microbiol Resour Announc.

[26] Kassinen A., Krogius-Kurikka L., Mäkivuokko H. (2007). The fecal microbiota of irritable bowel syndrome patients differs significantly from that of healthy subjects. Gastroenterology.

[27] Karlsson F.H., Fåk F., Nookaew I. (2012). Symptomatic atherosclerosis is associated with an altered gut metagenome. Nat Commun.

[28] Ferrocino I., Ponzo V., Gambino R. (2018). Changes in the gut microbiota composition during pregnancy in patients with gestational diabetes mellitus (GDM). Sci Rep.

[29] Chen J., Wright K., Davis J.M. (2016). An expansion of rare lineage intestinal microbes characterizes rheumatoid arthritis. Genome Med.

[30] Gomez-Arango L.F., Barrett H.L., Wilkinson S.A. (2018). Low dietary fiber intake increases Collinsella abundance in the gut microbiota of overweight and obese pregnant women. Gut Microb.

[31] Kim S., Goel R., Kumar A. (2018). Imbalance of gut microbiome and intestinal epithelial barrier dysfunction in patients with high blood pressure. Clin Sci.

[32] Jie Z., Xia H., Zhong S.-L. (2017). The gut microbiome in atherosclerotic cardiovascular disease. Nat Commun.

[33] Zuo K., Li J., Li K. (2019). Disordered gut microbiota and alterations in metabolic patterns are associated with atrial fibrillation. GigaScience.

[34] Cui X., Ye L., Li J. (2018). Metagenomic and metabolomic analyses unveil dysbiosis of gut microbiota in chronic heart failure patients. Sci Rep.

[35] Dziarski R., Park S.Y., Kashyap D.R., Dowd S.E., Gupta D. (2016). Pglyrp-regulated gut microflora Prevotella falsenii, Parabacteroides distasonis and Bacteroides eggerthii enhance and Alistipes finegoldii attenuates colitis in mice. PLoS One.

[36] Campion D., Giovo I., Ponzo P., Saracco G.M., Balzola F., Alessandria C. (2019). Dietary approach and gut microbiota modulation for chronic hepatic encephalopathy in cirrhosis. World J Hepatol.

[37] Andoh A. (2016). Physiological role of gut microbiota for maintaining human health. Digestion.

[38] Arpaia N., Campbell C., Fan X. (2013). Metabolites produced by commensal bacteria promote peripheral regulatory T cell generation. Nature.

[39] Polansky O., Sekelova Z., Faldynova M., Sebkova A., Sisak F., Rychlik I. (2016). Important metabolic pathways and biological processes expressed by chicken cecal microbiota. Appl Environ Microbiol.

[40] Oliphant K., Allen-Vercoe E. (2019). Macronutrient metabolism by the human gut microbiome: major fermentation by-products and their impact on host health. Microbiome.

[41] Parker B.J., Wearsch P.A., Veloo A.C.M., Rodriguez-Palacios A. (2020). The genus Alistipes: gut bacteria with emerging implications to inflammation, cancer, and mental health. Front Immunol.

[42] Jiang W., Wu N., Wang X. (2015). Dysbiosis gut microbiota associated with inflammation and impaired mucosal immune function in intestine of humans with non-alcoholic fatty liver disease. Sci Rep.

[43] Meador J.P., Caldwell M.E., Cohen P.S., Conway T. (2014). Escherichia coli pathotypes occupy distinct niches in the mouse intestine. Infect Immun.

[44] Porter S.B., Johnston B.D., Kisiela D., Clabots C., Sokurenko E.V., Johnson J.R. (2022). Bacteriophage cocktail and microcin-producing probiotic Escherichia coli protect mice against gut colonization with multidrug-resistant Escherichia coli sequence type 131. Front Microbiol.

