# Supplementary material for: Gut microbiome predictors of Escherichia coli sequence type 131 colonization and loss
Source: eBioMedicine. 2023 Dec 13;99:104909. doi: 10.1016/j.ebiom.2023.104909 (PMC10758731; doi:10.1016/j.ebiom.2023.104909)
Supplement: Supplementary Figures and Table [file mmc1.docx]

**Supplementary Materials: Bacterial predictors of *Escherichia coli* sequence type 131 colonization and loss**

Contents

[**Supplemental Table S1.** Prevalence and mean proportional abundance of genera in cross-sectional analysis, stratified by *Escherichia* and ST131 subclone categories. 2](#_Toc103697000)

[**Supplemental Figure 1.** Phylum-level proportional abundance of gut microbiome taxa among participants at baseline, ordered by Actinobacteria proportional abundance and stratified by ST131 and *Escherichia/Shigella* status. 4](#_Toc103697001)

[**Supplemental Figure 2.** Genus-level proportional abundance of gut microbiome taxa among participants at baseline, ordered by *Collinsella* proportional abundance and stratified by ST131 and *Escherichia/Shigella* status. 5](#_Toc103697002)

[**Supplemental Figure 3.** Phylum-level proportional abundance of gut microbiome taxa among participants at baseline, stratified by ST131 carriage status. 6](#_Toc103697003)

[**Supplemental Figure 4.** Phylum-level alpha diversity (Shannon index) at baseline, stratified by ST131 carriage status. 7](#_Toc103697004)

[**Supplemental Table S2.** Genera associated^a^ with *H*30R carriage compared with ST131-negative status across longitudinally collected samples from persistent negative or sustained loss participants. 8](#_Toc103697005)

# **Supplemental Table S1.** Prevalence and mean proportional abundance of genera in cross-sectional analysis, stratified by *Escherichia* and ST131 subclone categories.

| **Phylum or *genus*** | **Prevalence^a^, percent** | | | | | **Proportional Abundance^b^, mean (SD)** | | | | |
| --- | --- | --- | --- | --- | --- | --- | --- | --- | --- | --- |
|  | **Non-ST131** | | **ST131-positive** | | | **Non-ST131** | | **ST131-positive** | | |
|  | ***Escherichia* -negative** | ***Escherichia*-positive** | **ST131 non-*H*30R** | ***H*30R, non-Rx** | ***H*30Rx** | ***Escherichia*-negative** | ***Escherichia*-positive** | **ST131 non-*H*30R** | ***H*30R, non-Rx** | ***H*30Rx** |
|  | **n=55** | **n=386** | **n=29** | **n=38** | **n=11** | **n=55** | **n=386** | **n=29** | **n=38** | **n=11** |
| **Actinobacteria** | 87.3 | 95.3 | 100 | 92.1 | 100 | 1.3 (1.6) | 3.3 (4.8) | 4.3 (3.6) | 4.4 (4.1) | 6.7 (4.0) |
| *Asaccharobacter** | 5.5 | 28.2 | 48.3 | 42.1 | 54.6 | 0.0 (0.0) | 0.1 (0.1) | 0.1 (0.2) | 0.1 (0.2) | 0.2 (0.2) |
| *Bifidobacterium* | 52.7 | 63.2 | 72.4 | 65.8 | 81.8 | 0.6 (1.3) | 1.0 (2.5) | 0.8 (1.2) | 0.7 (1.3) | 0.8 (1.0) |
| *Collinsella* | 41.8 | 70.2 | 89.7 | 71.1 | 100 | 0.2 (0.6) | 1.3 (2.7) | 1.9 (2.3) | 1.7 (2.2) | 4.1 (3.4) |
| *Eggerthella* | 47.3 | 47.4 | 72.4 | 55.3 | 54.6 | 0.2 (0.3) | 0.2 (0.4) | 0.7 (1.0) | 0.7 (1.3) | 0.2 (0.4) |
| *Gardnerella* | 1.8 | 7.8 | 37.9 | 18.4 | 18.2 | 0.0 (0.0) | 0.0 (0.3) | 0.1 (0.3) | 0.2 (1.2) | 0.5 (1.5) |
| *Gordonibacter* | 20.0 | 33.7 | 55.2 | 50.0 | 63.6 | 0.0 (0.1) | 0.3 (1.1) | 0.5 (1.2) | 0.6 (1.4) | 0.4 (0.6) |
| **Bacteroidetes** | 100 | 100 | 100 | 100 | 100 | 37.2 (21.9) | 29.6 (14.4) | 24.5 (13.0) | 29.7 (12.2) | 28.2 (15.4) |
| *Alistipes* | 87.3 | 88.1 | 89.7 | 76.3 | 81.8 | 3.7 (4.1) | 2.4 (3.7) | 1.3 (1.4) | 2.0 (3.7) | 2.5 (2.5) |
| *Bacteroides* | 98.2 | 99.7 | 100 | 100 | 100 | 28.4 (21.9) | 23.3 (13.3) | 20.4 (11.3) | 23.9 (12.3) | 21.5 (11.8) |
| **Firmicutes** | 96.4 | 100 | 100 | 100 | 100 | 45.6 (26.1) | 38.9 (16.8) | 39.2 (16.5) | 41.0 (13.5) | 45.0 (10.5) |
| *Enterococcus* | 38.2 | 43.3 | 55.2 | 52.6 | 45.5 | 1.5 (4.5) | 0.7 (2.8) | 4.5 (10.5) | 2.3 (6.8) | 0.4 (0.7) |
| *Lactobacillus* | 18.2 | 35.5 | 62.1 | 68.4 | 63.6 | 0.2 (1.0) | 0.3 (1.2) | 1.0 (3.0) | 1.7 (3.9) | 0.5 (1.1) |
| *Streptococcus* | 60.0 | 68.7 | 96.6 | 81.6 | 90.9 | 1.4 (3.0) | 1.2 (4.0) | 1.5 (2.0) | 1.1 (1.9) | 6.4 (10.2) |
| *Weissella* | 1.8 | 2.3 | 13.8 | 7.9 | 0 | 0.1 (0.6) | 0.0 (0.2) | 0.0 (0.1) | 0.0 (0.2) | - |
| **Fusobacteria** | 7.3 | 13.7 | 13.8 | 13.2 | 18.2 | 0.1 (0.6) | 0.2 (1.3) | 0.1 (0.2) | 0.2 (1.1) | 0.0 (0.0) |
| **Lentisphaerae** | 5.5 | 10.9 | 0 | 5.3 | 18.2 | 0.0 (0.0) | 0.0 (0.1) | - | 0.0 (0.0) | 0.0 (0.0) |
| **Proteobacteria** | 83.6 | 100 | 100 | 100 | 100 | 13.8 (19.5) | 26.3 (20.0) | 31.3 (18.6) | 22.9 (16.4) | 19.1 (15.0) |
| *Escherichia* | 0 | 100 | 100 | 100 | 90.9 | - | 21.3 (19.6) | 22.8 (16.8) | 17.7 (15.4) | 17.4 (15.9) |
| *Oxalobacter** | 1.8 | 4.2 | 3.5 | 18.4 | 9.1 | 0.0 | 0.0 | 0.0 | 0.0 | 0.0 |
| **Synergistetes** | 12.7 | 13.0 | 0 | 15.8 | 36.4 | 0.1 (0.3) | 0.1 (0.3) | - | 0.7 (4.2) | 0.2 (0.8) |
| **Verrucomicrobia** | 41.8 | 53.4 | 34.5 | 42.1 | 36.4 | 2.0 (4.1) | 1.2 (3.3) | 0.2 (0.8) | 0.4 (1.1) | 0.1 (0.2) |
| *Akkermansia* | 41.8 | 53.4 | 34.5 | 42.1 | 36.4 | 2.0 (4.1) | 1.2 (3.3) | 0.2 (0.8) | 0.4 (1.1) | 0.1 (0.2) |

*Genetic near-neighbor of the given taxon

a. percent of participants in a group with a given taxon

b. mean proportional contribution of each taxa to the gut microbiome of each participant, averaged across each group. PerMANOVA comparing proportional abundance at the phylum level across groups (Non-ST131, ST131 non-H30R, H30R non-Rx, H30Rx), p-value=0.158

# **Supplemental Figure 1.** Phylum-level proportional abundance of gut microbiome taxa among participants at baseline, ordered by Actinobacteria proportional abundance and stratified by ST131 and *Escherichia/Shigella* status.

**
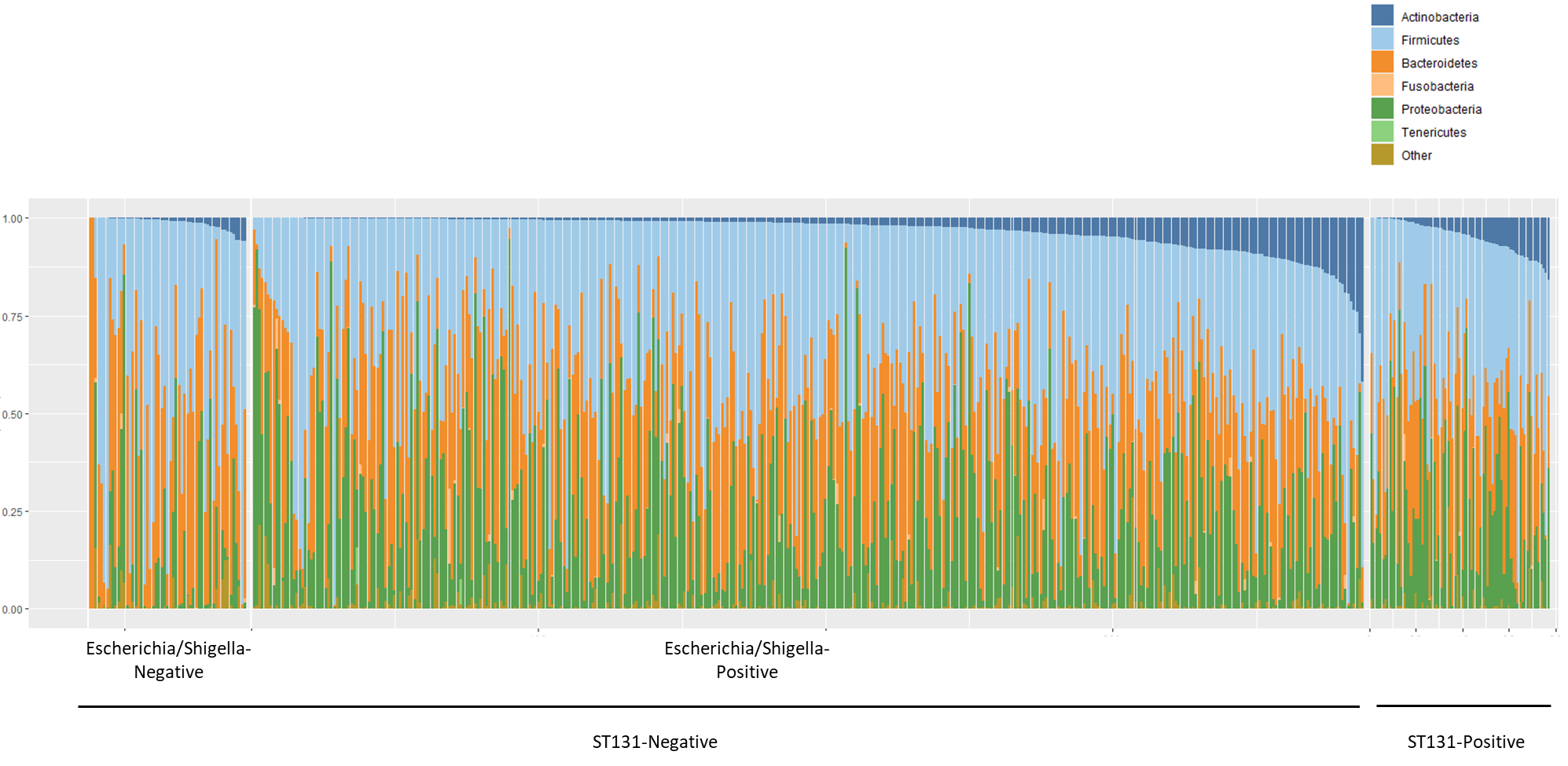
**

# **Supplemental Figure 2.** Genus-level proportional abundance of gut microbiome taxa among participants at baseline, ordered by *Collinsella* proportional abundance and stratified by ST131 and *Escherichia/Shigella* status.

**
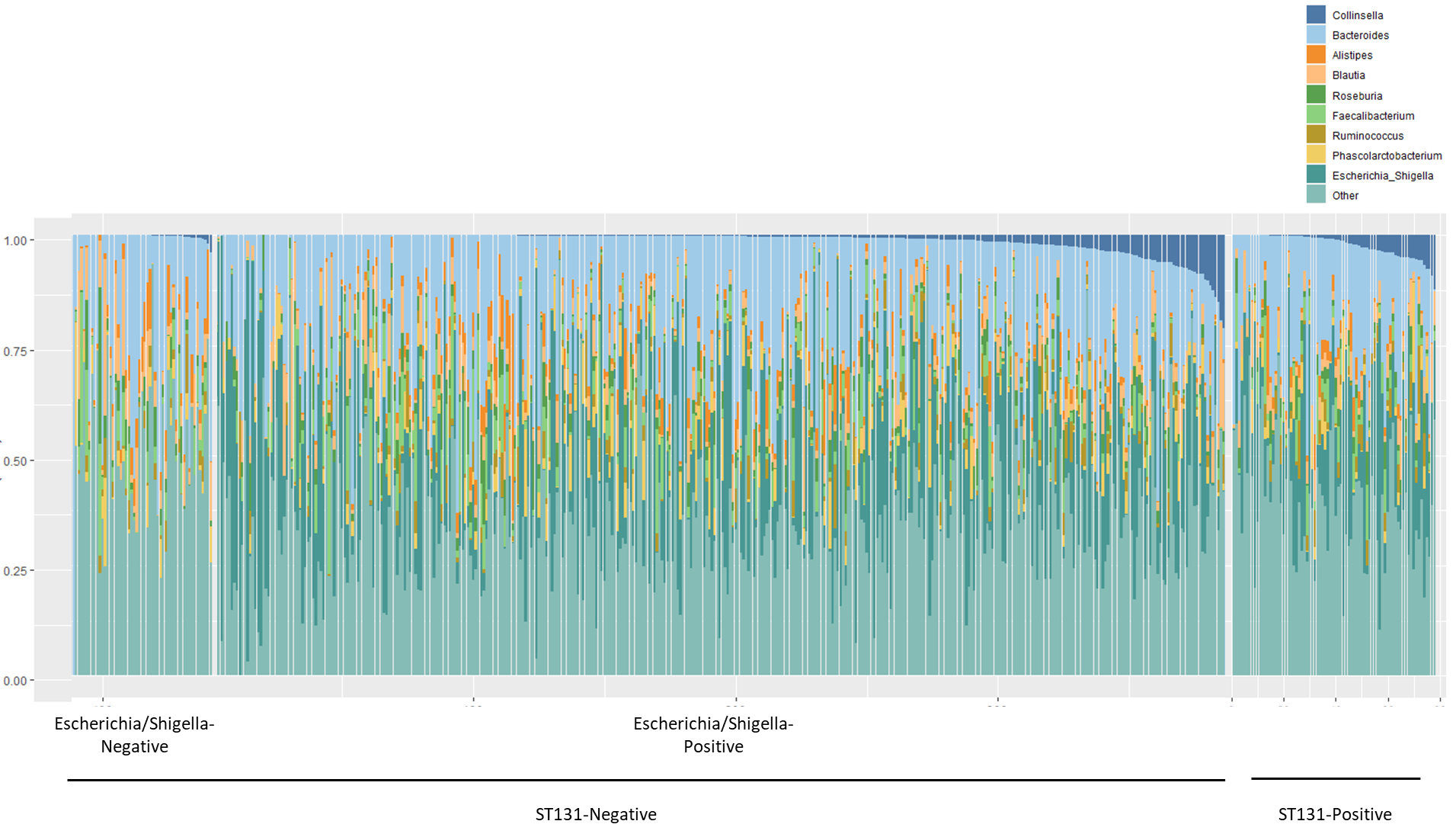
**

# **Supplemental Figure 3.** Phylum-level proportional abundance of gut microbiome taxa among participants at baseline, stratified by ST131 carriage status.

**
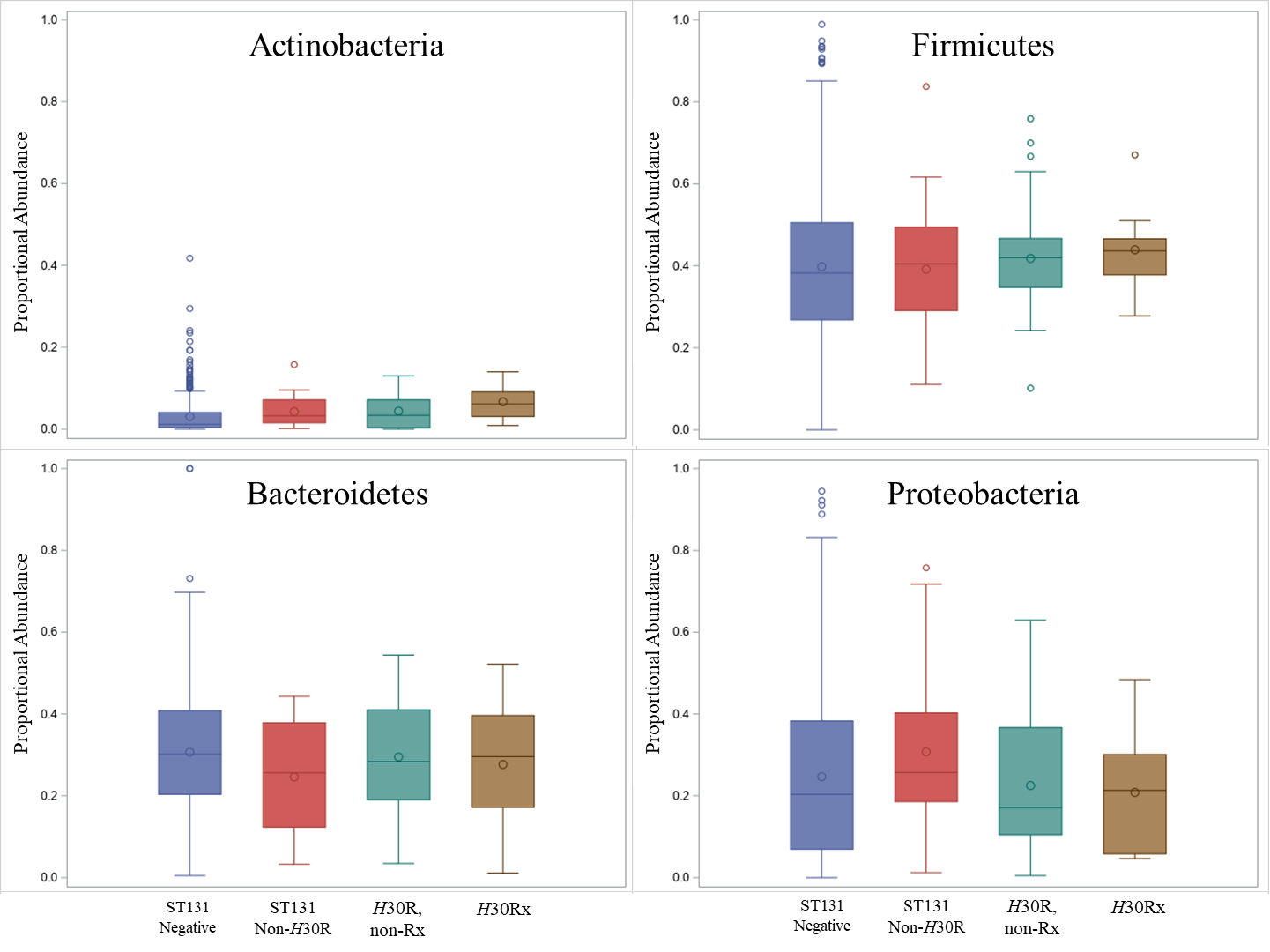
**

Box-and-whisker plot of each phyla with median values (line) of each by ST131 carriage status. Whiskers represent maximum and minimum within 1.5 times the inner quartile range, and boxes represent the 25th to 75th percentiles.

# **Supplemental Figure 4.** Phylum-level alpha diversity (Shannon index) at baseline, stratified by carriage status of A) ST131-negative and ST131-H30R subclones, and B) ST131-negative and ST131-positive sequence types.


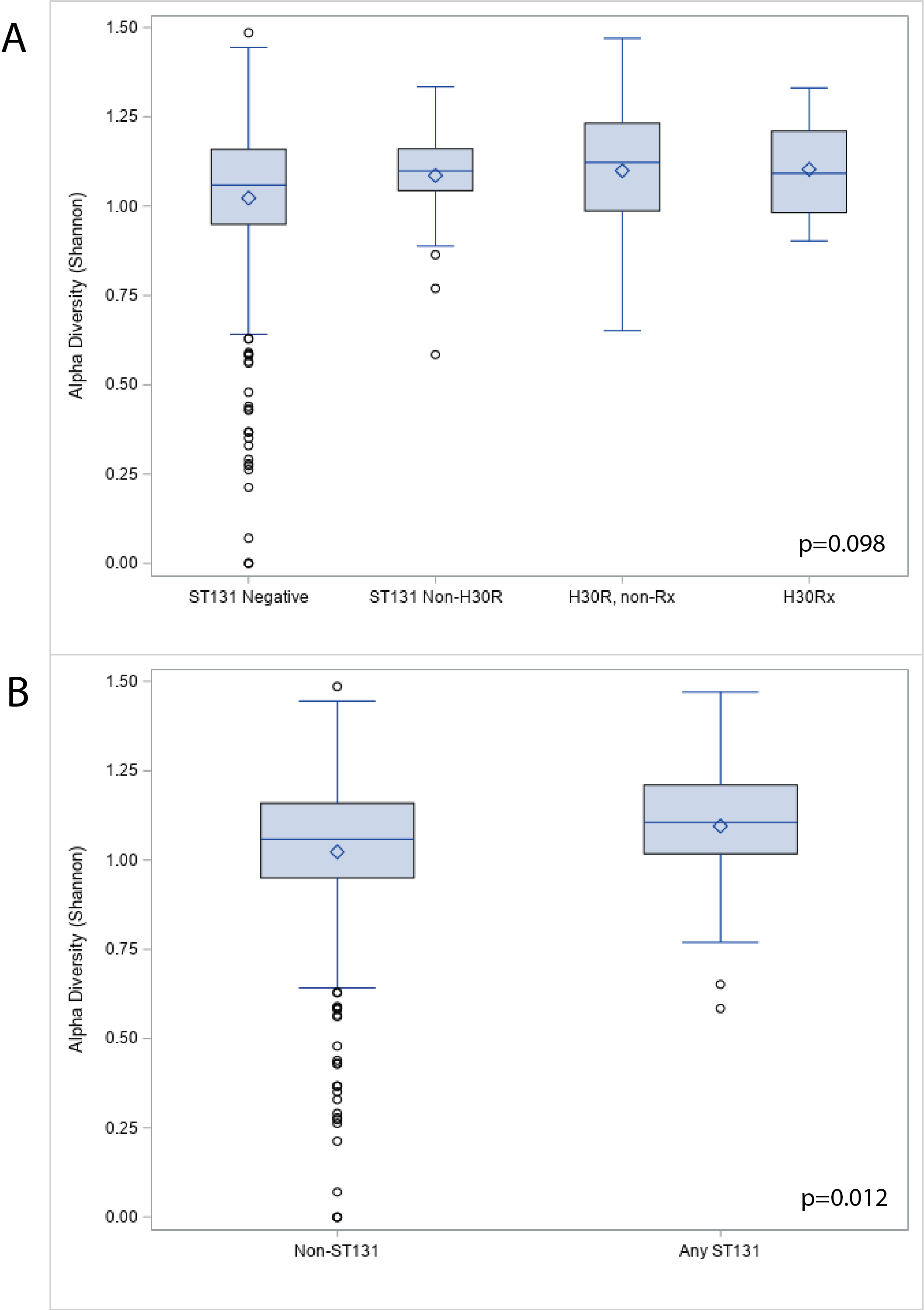


# **Supplemental Table S2.** Genera associated^a^ with *H*30R carriage compared with ST131-negative status across longitudinally collected samples from exposed sustained negative and sustained loss participants.

|  | **Model Estimate** | **Standard Error** | **t value** | **Probability > \|t\|** |
| --- | --- | --- | --- | --- |
| **Actinobacteria** |  |  |  |  |
| *Asaccharobacter** | -109.5 | 188.1 | -0.58 | 0.56 |
| *Bifidobacterium* | -25.1 | 42.3 | -0.59 | 0.55 |
| *Collinsella* | 31.7 | 14.9 | 2.13 | **0.035** |
| *Eggerthella* | 43.6 | 45.1 | 0.97 | 0.34 |
| *Gardnerella* | -6.6 | 64.4 | -0.10 | 0.92 |
| *Gordonibacter* | -5.1 | 23.5 | -0.22 | 0.83 |
| **Bacteroidetes** |  |  |  |  |
| *Alistipes* | -66.3 | 23.4 | -2.83 | **0.005** |
| *Bacteroides* | 0.4 | 2.7 | 0.15 | 0.89 |
| **Firmicutes** |  |  |  |  |
| *Enterococcus* | -0.5 | 4.3 | -0.11 | 0.92 |
| *Lactobacillus* | 4.9 | 22.3 | 0.22 | 0.83 |
| *Streptococcus* | 37.1 | 23.3 | 1.59 | 0.11 |
| *Weissella* | -99.1 | 221.6 | -0.45 | 0.66 |
| **Proteobacteria** |  |  |  |  |
| *Escherichia* | 0.2 | 2.0 | 0.08 | 0.93 |
| *Oxalobacter** | 2929.2 | 2474.3 | 1.18 | 0.24 |
| **Verrucomicrobia** |  |  |  |  |
| *Akkermansia* | 19.5 | 28.9 | 0.67 | 0.50 |

* Genetic near-neighbor of a given taxon

a. Evaluated using a mixed effects logistic regression model using taxa proportional abundance

**Supplemental Table S3**. Prevalence and mean proportional abundance of species in cross-sectional analysis, by individuals with differing categories of *E. coli* ST131 and ST131 subclone (H30R and non-H30R) carriage status

| ***Genus*** or *species* | **Prevalence^b^, column percent** | | | | **P value** | | **Proportional abundance^c^, mean (SD)** | | | | **P value** | |
| --- | --- | --- | --- | --- | --- | --- | --- | --- | --- | --- | --- | --- |
|  | **A. Non-ST131** | **B. Any ST131** | **C. ST131, non-*H*30R** | **D. ST131, *H*30R** | **A vs B** | **A v D** | **A. Non-ST131** | **B. Any ST131** | **C. ST131, non-*H*30R** | **D. ST131, *H*30R** | **A vs B** | **A v D** |
|  | **N = 441** | **N = 78** | **N = 29** | **N = 49** |  |  | **N = 441** | **N = 78** | **N = 29** | **N = 49** |  |  |
| ***Collinsella*** | 67 | 82 | 90 | 78 | **0.007** | 0.12 | 1.1 (2.5) | 2.1 (2.5) | 1.9 (2.3) | 2.3 (2.7) | **< 0.001** | **0.001** |
| *C. aerofaciens* | 53 | 79 | 86 | 75 | **<0.001** | **0.004** | 0.8 (2.1) | 1.8 (2.4) | 1.6 (2.2) | 2 (2.5) | **<0.001** | **<0.001** |
| *C. intestinalis* | 15 | 26 | 31 | 23 | **0.01** | 0.13 | 0.1 (1.1) | 0.3 (1) | 0.3 (1) | 0.3 (1) | **0.01** | 0.11 |
| *C. tanakaei* | 4 | 5 | 3 | 6 | 0.52 | 0.38 | 0.1 (0.7) | 0 (0.1) | 0.0 (0) | 0.0 (0.2) | 0.52 | 0.38 |
| ***Alistipes*** | 88 | 82 | 90 | 78 | 0.15 | **0.04** | 2.6 (3.8) | 1.8 (2.9) | 1.2 (1.4) | 2.1 (3.5) | **0.01** | 0.08 |
| *A. putredinis* | 57 | 52 | 48 | 54 | 0.44 | 0.73 | 0.6 (0.9) | 0.5 (1) | 0.4 (0.9) | 0.5 (1.1) | 0.09 | 0.28 |
| *A. finegoldii* | 34 | 34 | 28 | 38 | 0.99 | 0.60 | 0.3 (0.8) | 0.3 (1.1) | 0.1 (0.3) | 0.3 (1.3) | 0.69 | 0.94 |
| *A. onderdonkii* | 49 | 48 | 41 | 52 | 0.92 | 0.66 | 0.9 (2.9) | 0.5 (1.2) | 0.3 (0.6) | 0.6 (1.4) | 0.61 | 0.94 |
| *A. shahii* | 63 | 61 | 55 | 65 | 0.72 | 0.85 | 0.8 (1.6) | 0.5 (0.9) | 0.5 (0.8) | - 1. (0.9) | 0.39 | 0.93 |
| *A. timonensis* | 5 | 8 | 3 | 10 | 0.36 | 0.14 | 0.1 (0.4) | 0.0 (0.2) | 0.0 (0) | 0.0 (0.2) | 0.40 | 0.16 |
| ***Lactobacillus*** | 33 | 65 | 62 | 67 | **< 0.001** | **< 0.001** | 0.3 (1.2) | 1.3 (3.3) | 1.0 (3.0) | 1.5 (3.5) | **< 0.001** | **< 0.001** |
| *L. crispatus* | 8 | 19 | 28 | 15 | **0.001** | 0.09 | 0.0 (0.1) | 0.1 (1) | 0.3 (1.6) | 0.0 (0.2) | **0.001** | 0.10 |
| *L. gasseri* | 6 | 14 | 17 | 13 | **0.02** | **0.13** | 0.0 (0.3) | 0.1 (0.4) | 0.1 (0.2) | 0.1 (0.5) | **0.02** | 0.11 |
| *L. reuteri* | 3 | 14 | 14 | 15 | **<0.001** | **<0.001** | 0.0 (0.3) | 0.1 (0.2) | 0.0 (0.1) | 0.1 (0.3) | **<0.001** | **<0.001** |
| *L. salivarius* | 3 | 9 | 7 | 10 | **0.01** | **0.01** | 0.0 (0.2) | 0.1 (0.6) | 0.0 (0) | 0.2 (0.7) | **0.01** | **0.01** |
| *L. animalis* | 1 | 5 | 7 | 4 | **0.004** | **0.04** | 0.0 (0.1) | 0.2 (1.6) | 0.0 (0) | 0.3 (2) | **0.004** | **0.04** |
| *L. iners* | 11 | 48 | 48 | 48 | **<0.001** | **<0.001** | 0.0 (0.4) | 0.4 (1.7) | 0.4 (1.4) | 0.4 (1.9) | **<0.001** | **<0.001** |
| *L. mucosae* | 1 | 4 | 7 | 2 | **0.01** | 0.22 | 0.0 (0.2) | 0.0 (0.3) | 0.0 (0.1) | 0.1 (0.4) | **0.01** | 0.22 |
| ***Streptococcus*** | 68 | 88 | 97 | 84 | **< 0.001** | **0.02** | 1.2 (3.9) | 2.0 (4.5) | 1.5 (2.0) | 2.3 (5.4) | **< 0.001** | **0.001** |
| *S. anginosus* | 9 | 19 | 28 | 15 | **0.01** | 0.20 | 0.1 (1.1) | 0.1 (0.2) | 0.1 (0.2) | 0.1 (0.2) | **0.01** | 0.18 |
| *S. infantarius* | 5 | 8 | 14 | 4 | 0.42 | 0.71 | 0.1 (2) | 0.1 (0.7) | 0.2 (1.1) | 0.0 (0) | 0.43 | 0.75 |
| *S. mitis* | 4 | 9 | 7 | 10 | 0.09 | 0.07 | 0.3 (2.7) | 0.6 (3.9) | 0.1 (0.3) | 0.9 (4.9) | 0.09 | 0.08 |
| *S. mutans* | 6 | 25 | 21 | 27 | **<0.001** | **<0.001** | 0.0 (0.1) | 0.0 (0.1) | 0.0 (0.0) | 0.1 (0.2) | **<0.001** | **<0.001** |
| *S. salivarius* | 44 | 75 | 86 | 69 | **<0.001** | **0.001** | 0.0 (0.2) | 0.1 (0.3) | 0.1 (0.2) | 0.1 (0.4) | **<0.001** | **<0.001** |
| *S. sinensis* | 18 | 39 | 38 | 40 | **<0.001** | **0.001** | 0.5 (1.7) | 0.9 (1.5) | 0.8 (1.3) | 1.0 (1.6) | **<0.001** | **0.001** |

Prevalence and mean proportional abundance of genera and species, by *E. coli* ST131 carriage status (no ST131, any ST131, and ST131 subsets of non-*H*30R and any *H*30R). Species that had at least 0.1% proportional abundance and 1% overall prevalence were included.
